# Influence of Isometric Exercise Combined With Electromyostimulation on Inflammatory Cytokine Levels, Muscle Strength, and Knee Joint Function in Elderly Women With Early Knee Osteoarthritis

**DOI:** 10.3389/fphys.2021.688260

**Published:** 2021-07-13

**Authors:** Sunhee Park, Sukyung Min, Si-Hwa Park, Jaehyun Yoo, Yong-Seok Jee

**Affiliations:** ^1^Research Institute of Sports and Industry Science, Hanseo University, Seosan, South Korea; ^2^Department of Physical Education, Sahmyook University, Seoul, South Korea

**Keywords:** resistin, C-reactive protein, tumor necrosis factor-α, interleukin-6, Knee injury and Osteoarthritis Outcome Score (KOOS), electrical muscular stimulation

## Abstract

**Background and Objectives:** Muscle strengthening exercise is suggested to beneficial for patients with knee osteoarthritis (OA) and electrical muscular stimulation is reported to be effective in improvement of muscle strength. This study examined whether isometric exercise combined with whole body-electromyostimulation (WB-EMS) can improve serum cytokine levels, muscle strength, and knee function in elderly women with early knee OA.

**Materials and Methods:** This randomized controlled study included 75 participants assigned into three groups: the control group (CON), isometric exercise group (ISOM), and isometric exercise and electromyostimulation group (ISOM + EMS). The two exercise groups performed their respective programs for 8 weeks, 3 days a week, 30 min a day. The main exercises for both groups were performed continuously during the 20 min in an alternation of a 6-s contraction with a 4-s break. At pre- and post-intervention, anthropometric variables, muscle strength, Knee Injury and Osteoarthritis Outcome Score (KOOS), and blood sampling for biomarkers including interleukin-6, tumor necrosis factor-α, C-reactive protein, and resistin were performed.

**Results:** All variables at pre-intervention showed no significant differences among the three groups. However, there were significant differences between groups for body composition, muscle strength, KOOS subscale scores, and biomarkers. ISOM + EMS group resulted in a significant reduction in body weight, fat mass, fat percentage, inflammatory cytokine levels, and increased muscle strength. An ISOM + EMS group had the best KOOS score among all groups.

**Conclusion:** Isometric exercise combined with WB-EMS resulted in the best overall improvements in knee function and alleviating the pain and symptoms of patients with early knee OA. Further, reduced levels of inflammatory cytokines were observed. These non-pharmacologic, non-invasive interventions should be considered by healthcare specialists for elderly patients with early knee OA.

## Introduction

Knee osteoarthritis (OA), a degenerative joint disease, is predicted that the number of people affected with knee OA will continue to increase because of the aging of the population and the obesity epidemic (Heidari, 2011; Lespasio et al., 2017). The early diagnosis and proper treatment are important because of the increasing economic burden associated with OA (Samut et al., 2015). Although the pathophysiology of OA remains poorly understood, it has been reported that multifactorial factors such as genetics, age, obesity, smoking, joint injury, and metabolic dysfunction are involved in knee OA (Sandell, 2012; Lespasio et al., 2017). Modifiable risk factors, such as obesity and smoking, can be targeted for treatment (Lespasio et al., 2017).

Knee OA affects three compartments of the knee joint (medial, lateral, and patellofemoral joints) and interferes with daily life activities (Roos and Arden, 2016). It has been reported that people with knee OA experience atrophy of the muscles surrounding the knee joint. In particular, quadriceps muscle weakness is frequently found in patients with knee OA regardless of the presence of knee pain or muscular atrophy (Eyigor et al., 2004). This is because the quadriceps muscle is related to functional tasks, including standing up from a chair, climbing up and down the stairs, and walking on a level surface (Maly et al., 2006; Liikavainio et al., 2008; Alnahdi et al., 2012). Strengthening muscles related to the knee joint through exercise has been suggested to have beneficial effects in patients with knee OA such as significant reduction of knee pain and improvement of knee function (Anwer and Alghadir, 2014). Quadriceps muscle strengthening exercises are reported to be more effective when combined with other electrotherapy modalities or the Russian electrical stimulation (Kus and Yeldan, 2019).

For decades, electromyostimulation (EMS) or electrical muscular stimulation has been used for the treatment of sports-related injuries, post-exercise recovery, and athletic performance enhancement (Teschler and Mooren, 2019). Especially, whole body-EMS (WB-EMS) has been reported to be effective in improving the muscle strength of deconditioned subjects (Kemmler et al., 2017), and suggested to be a time-efficient, joint-friendly, and highly individualized exercise technology making it a good choice for older subjects (Brigatto et al., 2019). WB-EMS is also demonstrated to be a highly customizable option for people either unable or unmotivated to conduct intense (resistance) exercise protocols because leg-extensor strength and advanced lower extremity functions were found in older men after WB-EMS (Kemmler et al., 2018). Neuromuscular electrical stimulation is suggested to be effective for enhancing quadriceps muscle strength, knee pain, and physical function in patients with knee OA (Giggins et al., 2012). Moreover, previous studies have reported that the combined treatment of EMS with various interventions such as laser therapy (de Oliveira Melo et al., 2016) or volitional contraction (Paillard, 2008) had beneficial effects in patients with knee OA through improving the quadriceps strength, knee pain relief, and physical function.

Increased plasma levels of inflammatory cytokines such as tumor necrosis factor-alpha (TNF-α) and interleukin-6 (IL-6) have been reported in the elderly (Petersen and Pedersen, 2005). This low-grade inflammation is postulated to be involved in the pathological process of chronic conditions related to old age like knee OA (Santos et al., 2011). It has been suggested that regular exercise protects against diseases associated with chronic low-grade systemic inflammation (Petersen and Pedersen, 2005) and has anti-inflammatory effects in elderly women by decreasing serum cytokine levels (Ogawa et al., 2010). To our knowledge, there are only few studies on the combination of isometric exercise and electrical muscular stimulation in people with knee OA. Therefore, the aim of this randomized controlled study was to investigate the effects of isometric exercise combined with WB-EMS on elderly women with early knee OA over the course of time of eight weeks and compare the observed effects between groups through the measurements of serum cytokine levels, muscle strength, and knee function.

## Materials and Methods

### Participants and Study Design

In our study, we recruited participants aged between 61 and 79 years old with knee OA from the Seoul Seniors Tower in Korea. Subjects included were desirous to improve their OA and irregularly exercised for six months. The inclusion criteria included patients with (1) degenerative knee OA diagnosed by bilateral radiographic examination; (2) grade I or II knee OA levels (early knee OA); (3) age over 60 years; and (4) female sex. The radiographic examination was carried out a radiological technologist. Briefly, the anteroposterior, weight-bearing, short knee X-ray was obtained with the patient standing with the back of their knees in contact with the vertical cassette, and the result was graded by a radiologist. According to Kellgren-Lawrence grading, grade I demonstrates doubtful narrowing of the joint space with possible osteophyte formation, and grade II demonstrates possible narrowing of the joint space with definite osteophyte formation (Colebatch et al., 2009; Kohn et al., 2016). Participants were excluded if they had any of the following: deformity of the knee, hip, or back; central or peripheral nervous system involvement; administered any medications including steroids or intra-articular injection within previous three months, or previous surgery; pacemaker use; internal metallic materials; a history of impairment of a major organ system or a psychological disorder.

This prospective, randomized controlled study was conducted in accordance with the Declaration of Helsinki and was approved by the ethics committee (September 1, 2018, to August 31, 2019; 2-1040781-A-N-012021024HR). This study was registered at Korean Clinical Research Information Service (KCT0006037). Prior to the study, participants received detailed explanations regarding the study procedures, and written informed consent was obtained from all participants. A total of 92 participants were recruited through public announcements, exceeding the calculated effect size of the group for the experiment of 64.

**FIGURE 1:**
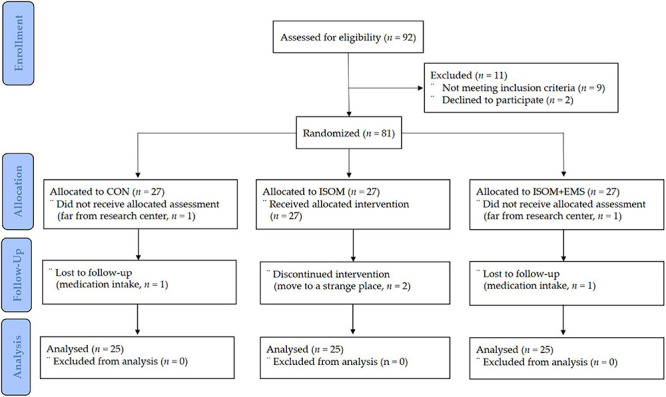
Participants’ allocation (consolidated standards for reporting of trials flow diagram). CON, control group; ISOM, isometric exercise group; ISOM + EMS, isometric exercise + electrostimulation group.

Subjects were assigned using random number tables and identification numbers upon recruitment. Eighty-one participants who met the inclusion criteria were randomly assigned to one of three groups: the control (CON, *n* = 27) group who was not provided with any intervention, the isometric exercise (ISOM, *n* = 27) group who performed isometric exercise while wearing a WB-EMS suit without electrical stimulation provided, and the isometric exercise and electromyostimulation (ISOM + EMS, *n* = 27) group who performed isometric exercise while wearing a WB-EMS suit with electrical stimulation provided. Throughout the study, two participants in the CON group were excluded from the allocation and follow-up phases. Two participants in the ISOM group were likewise excluded because they relocated during the follow-up phase. Two participants in the ISOM + EMS group were lost to follow-up during the follow-up phase. Finally, a total of 75 participants were included in the study as shown in Figure 1.

The participants were assessed at two different time points over eight weeks. The assessment was performed before the intervention period (pre-intervention) and after the intervention period (post-intervention). To prevent communication among the groups, the participants were classified according to their community areas. Moreover, the interventions and measurements were scheduled at different times to prevent communication between the CON, ISOM, and ISOM + EMS groups. The CON group was scheduled at 10:00 am, the ISOM group at 2:00 pm, and the ISOM + EMS group at 4:00 pm. At the beginning of the measurement for 1 maximal tolerance (1MT), only the ISOM + EMS group perceived the administered current from their suits. For the CON group, they gathered at the same center at the scheduled time and performed only meditation and light stretching by lying posture while wearing a suit for 30 min. Participant characteristics, which indicated homogeneity, are presented in Table 1.

**TABLE 1:** Demographic and baseline variables.

**Variables**	**CON (*n* = 25)**	**ISOM (*n* = 25)**	**ISOM + EMS (*n* = 25)**	**H^**†**^**	***p***
**Demographic, Body composition and Anthropometric**					
Age (y)	68.04 ± 4.16	66.88 ± 4.61	65.68 ± 3.24	4.390	0.111
Height (cm)	161.25 ± 7.31	161.54 ± 8.72	160.34 ± 8.14	0.247	0.884
Body weight (kg)	64.92 ± 9.84	64.20 ± 8.47	60.74 ± 8.12	2.380	0.304
Skeletal muscle mass (kg)	33.81 ± 6.32	33.80 ± 6.84	32.44 ± 5.36	0.435	0.804
Fat mass (kg)	28.29 ± 5.09	28.62 ± 4.14	27.31 ± 5.22	0.582	0.748
Fat percent (%)	30.17 ± 4.90	31.14 ± 6.18	32.17 ± 5.50	1.806	0.405
Basal metabolic rate (kcal)	1281.45 ± 144.32	1269.60 ± 143.40	1226.54 ± 125.94	1.613	0.446
**Biomerkers**					
IL-6 (pg/mL)	16.45 ± 6.17	16.56 ± 4.65	16.23 ± 6.51	0.008	0.996
TNF-α (pg/mL)	28.23 ± 6.98	28.26 ± 8.77	29.09 ± 11.20	0.181	0.914
CRP (pg/mL)	31.57 ± 13.64	32.08 ± 8.76	32.71 ± 9.43	1.080	0.583
RSTN (ng/mL)	5.62 ± 2.15	5.49 ± 1.69	5.47 ± 2.37	1.338	0.512
**Isokinetic torques of knee joint at 60 degree/second**					
PT of Flexor (Nm)	117.64 ± 29.95	119.44 ± 50.32	115.48 ± 38.78	0.235	0.889
PTBW of Flexor (Nm/kg)	1.84 ± 0.50	1.86 ± 0.71	1.91 ± 0.59	0.253	0.881
PT of Extensor (Nm)	139.64 ± 38.23	144.28 ± 35.28	138.96 ± 35.57	0.260	0.878
PTBW of Extensor (Nm/kg)	2.13 ± 0.38	2.25 ± 0.46	2.27 ± 0.40	1.201	0.549
**Knee Injury and Osteoarthritis Outcome Score (KOOS)**					
Pain	73.82 ± 7.36	72.40 ± 5.82	72.26 ± 4.19	0.160	0.923
Symptoms	74.24 ± 1.63	73.91 ± 1.49	74.21 ± 1.58	0.677	0.713
Activities of daily living	42.84 ± 4.88	40.26 ± 5.05	39.80 ± 5.87	5.194	0.074
Sports and recreation	26.00 ± 6.29	25.00 ± 2.89	27.40 ± 18.32	0.876	0.645
Knee-related quality of life	16.25 ± 4.03	18.50 ± 9.28	15.75 ± 11.28	2.896	0.235

*All values are expressed as mean ± standard deviation. ^†^ was analyzed using Kruskal-Wallis test for comparison between groups (CON, ISOM and ISOM + EMS). CON, control group; ISOM, isometric exercise group; ISOM + EMS, isometric exercise + electrostimulation group; IL-6, interleukin-6; TNF-α, tumor necrosis factor-α; CRP, C-reactive protein; RSTN, resistin.*

### Isometric Exercises With WB-EMS Administration

All participants were instructed to wear their WB-EMS suits and were blinded if electrical stimulation was provided. The ISOM and ISO + EMS groups performed an isometric exercise program wearing a suit with or without electromyostimulation. To protect the knee joint while improving the range of motion and strengthening the muscles around the knee joint, eight types of isometric movements were performed during the impulse phase as per the instructor’s direction. The isometric exercise used in this study was configured to fit the entire body where electrical stimulation enters the WB-EMS suit allowing exercise. The exercise regimen consists of abdomen crunches, bridge, leg raises, side planks, back extension, front planks, front lunges, and squats for 6 s with a 4-s break in between electrical stimulations. The EMS suits enabled the simultaneous activation of eight pairs of muscle groups (both upper legs, both upper arms, buttocks, abdomen, chest, lower back, upper back, and latissimus dorsi) with selectable intensities for each region, and the electrical strength of the suit was controlled via Bluetooth. The WB-EMS suits, manufactured by Miracle^®^ (Seoul, South Korea) composed of silicone conductive pads and wireless materials, were tailored to the participants’ body size.

According to previous studies, the stimulation frequency was set at 85 Hz, the impulse width at 350 μs, the impulse rise as a rectangular application, and electrostimulation intensities relative to the maximal ratings of perceived exertion scale, which is 1MT, similar to calculating the maximal voluntary contraction as one maximal repetition (Gondin et al., 2005; Micke et al., 2018; Berger et al., 2019; Kim and Jee, 2020). Each 1MT of the upper and lower body of the ISOM + EMS group was determined as the time when the electrical stimulation received through the WB-EMS suit could no longer be tolerated and was requested to be stopped. The electromyostimulation for 1MT was gradually increased from a low stimulation current to prevent surprising the participants or causing discomfort. The level of 1MT for each individual was stored with a Bluetooth device and also recorded as RPE. The percentage of maximal tolerance was obtained through the ratings of perceived exertion (RPE) scale ranging from 6 to 20 as shown in Table 2, where the lowest score (6) indicated “no exertion at all” and the highest score (20) indicated “maximal exertion” (Borg, 1982).

**TABLE 2:** Recorded 1MT and estimated EMS intensities based on RPE in an ISOM + EMS group.

**Participants**	**Recorded 1MT**	**Estimated intensities**		
		**60% 1MT**	**70% 1MT**	**80% 1MT**
1	18.00	11.00	13.00	14.00
2	19.00	11.00	13.00	15.00
3	17.00	10.00	12.00	14.00
4	18.00	11.00	13.00	14.00
5	19.00	11.00	13.00	15.00
6	16.00	10.00	11.00	13.00
7	17.00	10.00	12.00	14.00
8	17.00	10.00	12.00	14.00
9	18.00	11.00	13.00	14.00
10	19.00	11.00	13.00	15.00
11	19.00	11.00	13.00	15.00
12	20.00	12.00	14.00	16.00
13	17.00	10.00	12.00	14.00
14	17.00	10.00	12.00	14.00
15	18.00	11.00	13.00	14.00
16	19.00	11.00	13.00	15.00
17	17.00	10.00	12.00	14.00
18	18.00	11.00	13.00	14.00
19	19.00	11.00	13.00	15.00
20	17.00	10.00	12.00	14.00
21	17.00	10.00	12.00	14.00
22	18.00	11.00	13.00	14.00
23	19.00	11.00	13.00	15.00
24	19.00	11.00	13.00	15.00
25	19.00	11.00	13.00	15.00
Mean ± SD	18.04 ± 1.02	10.82 ± 0.61	12.63 ± 0.71	14.43 ± 0.82

*1MT, maximal tolerance; ISOM + EMS, isometric exercise + electrostimulation group; RPE, rating of perceived exertion.*

After determining the 1MT, the participants in the ISOM + EMS group were assigned to 60% of 1MT from the baseline to week 2, 70% of 1MT from week 3 to week 5, and 80% of 1MT from week 6 to week 8. As shown in Table 2, the RPE levels corresponding to 60%, 70% and 80% based on 1MT of all subjects assigned to the ISOM + EMS group were estimated. All participants were asked to express the difficulty level of the isometric exercise while wearing a WB-EMS suit. An assistant recorded the degree of RPE every 5 min. The estimated RPE level by three exercise intensities and the recorded RPE levels when isometric exercise was actually performed were compared and analyzed as shown in Table 3. The RPE levels reported when performing isometric exercise according to three exercise intensities in two exercise groups (ISOM and ISOM + EMS) were comparatively analyzed as shown in Table 4.

**TABLE 3:** Comparison result between estimated RPE level and measured RPE level during actual exercise for three exercise intensities per 5 min in the ISO + EMS group.

		**Paired *t*-test**	**Paired differences**			
**Minute**	**Intensity**	**mean ± SD**	**mean ± SD**	**95% CI**	**t^**†**^**	**Z^**‡**^**
	Estimated 60% 1MT	10.82 ± 0.61				
M5	Recorded 60% 1MT	11.00 ± 0.71	−0.18 ± 0.76	[−0.49 ∼ 0.14]	–1.161	–0.232
M10	Recorded 60% 1MT	11.80 ± 0.82	−0.98 ± 1.04	[−1.41 ∼ -0.55]	−4.693***	−3.708***
M15	Recorded 60% 1MT	12.24 ± 0.83	−1.42 ± 1.19	[−1.91 ∼ -0.92]	−5.928***	−3.948***
M20	Recorded 60% 1MT	13.16 ± 1.03	−2.34 ± 0.89	[−2.70 ∼ -1.97]	−13.149***	−4.391***

	Estimated 70% 1MT	12.63 ± 0.71				
M5	Recorded 70% 1MT	13.04 ± 0.79	−0.41 ± 0.85	[−0.76 ∼−0.06]	−2.416*	−2.206*
M10	Recorded 70% 1MT	13.36 ± 1.11	−0.73 ± 1.17	[−1.21 ∼−0.25]	−3.136**	−2.698**
M15	Recorded 70% 1MT	14.28 ± 1.24	−1.65 ± 1.27	[−2.17 ∼−1.13]	−6.525***	−4.178***
M20	Recorded 70% 1MT	14.52 ± 1.29	−1.89 ± 1.55	[−2.53 ∼−1.25]	−6.117***	−4.034***

	Estimated 80% 1MT	14.43 ± 0.82				
M5	Recorded 80% 1MT	14.40 ± 0.65	0.03 ± 0.33	[−0.10 ∼ 0.17]	0.485	–0.133
M10	Recorded 80% 1MT	14.76 ± 0.83	−0.33 ± 1.07	[−0.77 ∼ 0.11]	–1.536	–1.167
M15	Recorded 80% 1MT	14.92 ± 1.12	−0.49 ± 1.62	[−1.16 ∼ 0.18]	–1.503	–1.820
M20	Recorded 80% 1MT	15.84 ± 1.57	−1.41 ± 1.71	[−2.11 ∼−0.70]	−4.114***	−3.380***

All values are expressed as mean ± standard deviation. ^†^ was analyzed using paired t-test between estimated percent 1MT and actually recorded percent 1MT. ^‡^ was analyzed using Wilcoxon signed-rank test for comparison between estimated percent 1MT and actually recorded percent 1MT. ***, and *** represent *p* < 0.05, *p* < 0.01 and *p* < 0.001, respectively. ISOM, isometric exercise group; ISOM + EMS, isometric exercise + electrostimulation group; M, minute; RPE, rating of perceived exertion.

**TABLE 4:** Comparison result of RPE levels every 5 min for three exercise intensities in two exercised groups.

**Relative intensity**	**Minute**	**Groups**		
		**ISOM**	**ISOM + EMS**	**Z^**†**^**
60%	M5	10.80 ± 0.65	11.00 ± 0.71	–0.954
	M10	11.72 ± 0.98	11.80 ± 0.82	–0.215
	M15	12.32 ± 0.90	12.24 ± 0.83	–0.298
	M20	12.80 ± 0.65	13.16 ± 1.03	–1.076
70%	M5	11.32 ± 0.69	13.04 ± 0.79	−5.543***
	M10	12.32 ± 0.80	13.36 ± 1.11	−3.477***
	M15	12.96 ± 1.24	14.28 ± 1.24	−3.311***
	M20	13.28 ± 1.02	14.52 ± 1.29	−3.302***
80%	M5	12.56 ± 0.87	14.40 ± 0.65	−5.672***
	M10	12.04 ± 0.79	14.76 ± 0.83	−6.081***
	M15	12.92 ± 1.04	14.92 ± 1.12	−4.815***
	M20	14.00 ± 1.22	15.84 ± 1.57	−3.823***

All values are expressed as mean ± standard deviation. ^†^ was analyzed using Mann-Whitney U test for comparison of variables between groups (ISOM and ISOM + EMS). *, ** and *** represent *p* < 0.05, *p* < 0.01 and *p* < 0.001, respectively. ISOM, isometric exercise group; ISOM + EMS, isometric exercise + electrostimulation group; M, minute.

**FIGURE 2:**
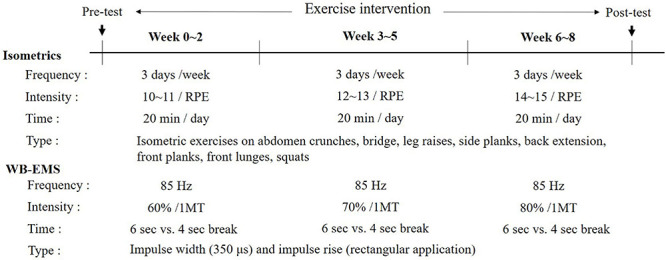
Flow chart regarding timing of outcome assessment and intervention.

For participants in the ISOM and ISOM + EMS groups, an instructor conducted the exercise program three times a week on two non-consecutive days (Monday, Wednesday, and Friday) to allow for a rest period of 48 h between each session. The ISOM and the ISOM + EMS groups in this study performed a total of 30 min of exercise per day. The main exercises for both groups were performed continuously during the 20 min in an alternation of a 6-s contraction with a 4-s break. Before and after the exercise intervention, full body stretch while lying down for warm-up and cool-down exercise was performed for 5 min without electrical stimulants, respectively. The static stretches were actively performed as follows: arms sweeps (15 s), floor hip flexor stretch (15 s ^∗^ 2), knees to chest (15 s ^∗^ 2), arms to knees (15 s ^∗^ 2), single knee rotation (15 s ^∗^ 2), double knee torso rotation (15 s ^∗^ 2), butterfly (15 s ^∗^ 2), Cobra with flexed elbows (15 s ^∗^ 2), kneel down (15 s), and then circles for knee (15 s), waist (15 s), shoulder (15 s), and neck circle (15 s). The flow chart regarding timing of outcome assessment and intervention of this study is as shown in Figure 2.

### Anthropometric Measurements

To measure body composition, all patients were weighed while wearing light clothes and no shoes. Bioelectrical impedance analysis was performed using the BMS 330 for height and InBody 320 (Biospace Co., Ltd., Seoul, South Korea) for body composition. This analyzer is a segmental impedance device that measures voltage drops in the upper and lower body. Eight tactile electrodes located in the palms, fingers, front soles, and rear soles were placed on the surfaces of the hands and feet. The precision of the repeated measurements expressed as the coefficient of variation was 0.6% for the percentage of fat mass (Zheng et al., 2009). This analyzer is a segmental impedance device in which the electrodes are made of stainless steel interfaces. The subjects stood upright by placing their bare feet on the foot electrodes and gripping the hand electrodes. For accurate inspection, food intake and water intake were prohibited for 4 h and 1 h before the test, respectively. Further, urination was encouraged 30 min prior measurements since it may affect body weight and body fluid measurements (Cha et al., 2014).

### Isokinetic Torque Measurement of the Knee Joint

An isokinetic dynamometer (HUMAC^®^ /NORM^TM^ Testing and Rehabilitation System, CSMI, MA, United States) was used to measure the isokinetic torque of the knee in this study. All subjects were enrolled in a stretching program and a warm-up program before the test. They performed at least four submaximal practices at test speed with each cycle comprising movements from complete knee extension to complete knee flexion. Participants were placed in the equipment’s adjustable seat, and the tested limb was placed and fixed using a Velcro strap on support over the quadriceps muscle, and the knee joint was positioned at 90° flexion. The testing apparatus was prepared, and the participants were positioned and stabilized uniformly. Each participant was tested at a 60°/s angular speed as recommended by Perrin (1993). Testing was performed on the involved side while sitting. The involved side was defined as a painful knee joint, and the more symptomatic knee was included in case of bilateral involvement. All tests were supervised by a trained researcher. The outcome measures consisted of the peak torque (PT) and body weight normalized peak torque (peak torque body weight, PTBW). All the data were analyzed by averaging the values measured from both sides.

### Arthritis Pain and Functional Status Measures

The Knee Injury and Osteoarthritis Outcome Score (KOOS) is a patient-reported outcome measure intended for young, middle-aged, and elderly adults with a knee injury and/or knee OA. It can be used to monitor disease course and outcomes following various interventions, such as surgical and pharmacologic treatments. It consists of 42 items in five separately scored subscales: pain (9 items), symptoms (7 items), activities of daily living (17 items), sports and recreation function (5 items), and knee-related quality of life (4 items). All items were scored from 0 (no problems) to 4 (extreme problems), and each of the five scores was calculated as the sum of the items included (actual raw score). Scores are transformed to a 0–100 scale according to formula, with zero representing extreme knee problems and 100 representing no knee problems commonly in orthopedic assessment scales and generic measures (Roos et al., 1998; Roos and Lohmander, 2003).

The KOOS has been validated in different populations who underwent surgical procedures due to knee complaints. Thus, it is reported to be a valid, reliable, and responsive self-administered instrument that can be used for the short-term and long-term follow-up of various types of knee injuries, including OA (Roos and Lohmander, 2003). Collins et al. (2016) also suggested that KOOS demonstrates adequate content validity, internal consistency, test-retest reliability, construct validity, and responsiveness for the age- and condition-relevant subscales. KOOS has been translated into various languages, including English, Swedish, German, and Danish (Roos and Lohmander, 2003). The Korean version of KOOS also demonstrated its validity, reliability, and responsiveness, which is considered to be a useful clinical metrology for patients with a knee injury (Seo et al., 2006).

### Biomarker Measurements

Blood samples were collected after fasting for 10 h or longer before the assessment and were collected using BD vacutainer tubes (Becton Dickinson, Franklin Lakes, NJ, United States). After resting for 15 min, 5 mL of blood was collected from the antecubital vein of the subjects with a disposable syringe by a medical laboratory technologist before and after the experiments. Blood samples were left at room temperature for 1 h and centrifuged at 1,000 rpm for 15 min for the serum.

IL-6, TNF-α, and C-reactive protein (CRP) were analyzed using an enzyme-linked immunosorbent assay (ELISA) kit (Cohesion Biosciences, London, United Kingdom). The minimum detectable dose of human IL-6 is typically less than 1 pg/mL. The IL-6 ELISA kit allows for the detection and quantification of endogenous levels of natural and/or recombinant human IL-6 proteins within the range of 3.9–250 pg/mL. TNF-α was allowed to clot in a serum separator tube at room temperature, was centrifuged at approximately 1000 × g for 15 min, and was immediately analyzed (Ko et al., 2014). The minimum detectable dose of human TNF-α is typically <7 pg/mL. The TNF-α ELISA Kit allows for the detection and quantification of endogenous levels of natural and/or recombinant human TNF-a proteins within the range of 15.6–1000 pg/mL. CRP was also analyzed using an ELISA kit. The minimum detectable dose of human CRP is usually < 10 pg/mL. The CRP ELISA kit allows for the detection and quantification of endogenous levels of natural and/or recombinant human CRP proteins within the range of 15.6–1000 pg/mL. Serum resistin (RSTN), an adipose tissue-specific secretory factor, was analyzed using an ELISA kit (Phoenix Pharmaceuticals, London, United Kingdom). The standard solution and sample for RSTN were added to a microplate coated with a specific RSTN monoclonal antibody and bound to RSTN (#EK-S-028-36) to form immobilized antibodies. Afterward, materials were removed by washing, and a biotinylated polyclonal antibody specific for biomarkers was added to each well. Unbound antibody-enzyme for biomarkers was removed by washing, and horseradish peroxidase was added to each well.

### Data Analysis

Microsoft Excel (Microsoft, Redmond, WA, United States) was used to analyze the data, expressed as mean ± standard deviation (SD). The sample size was determined using G^∗^Power v. 3.1.9.7 (Faul et al., 2009), considering *a priori* effect size of f^2^ (V) = 0.40 (large size effect), α error probability = 0.05, power (1-β error probability) = 0.80, number of groups = 3, numerator difference = 2, and number of covariates = 1. The total number of subjects from the three groups was calculated as 64 based on the original number calculated for this program, and 25 subjects were assigned to each group for the study. Statistical analysis was performed using IBM SPSS Statistics (ver. 25; IBM Corp., Armonk, NY, United States). Based on the results of the Shapiro–Wilk test, which was used to ascertain the normality of distribution for the examined variables, non-parametric statistical hypothesis tests were used to analyze the data. First, we compared between estimated RPE level and measured RPE level during actual exercise for three exercise intensities per 5 min in an ISOM + EMS group using the paired t-test and Wilcoxon signed-rank test. Then, we analyzed the RPE levels every 5 min for three exercise intensities in two exercised groups (ISOM and ISOM + EMS) using Mann-Whitney U test. Second, we compared the scores of variables at pre-intervention between the groups using the Kruskal–Wallis test to ascertain the differences between the groups at baseline (pre-intervention). The Wilcoxon signed-rank test was used to ascertain the changes in these variables in each group over time (pre- and post-intervention). Although there were no significant differences in the variables between the groups at baseline (pre-intervention time), a comparison of intervention effects was performed using the analysis of ranked covariance (ANCOVA) test with baseline measures of each variable as covariates. For the *post hoc* test, ranked ANCOVA was performed between the two groups and the Bonferroni adjusted *p*-value (< 0.05/6 = 0.0167) was used for multiple comparisons. All data are reported as mean ± standard deviation (SD), and the level of statistical significance chosen was *p* ≤ 0.05.

## Results

### Differences of RPE Intensity During Isometric Exercises With or Without Electrostimulation

Table 2 showed that the recorded 1MT and estimated EMS intensities (60, 70, and 80%) based on RPE in an ISOM + EMS group. Table 3 showed that comparison result between estimated RPE level and measured RPE level during actual isometric exercise for three exercise intensities per 5 min in the ISOM + EMS group. Table 4 showed that comparison result of RPE levels every 5 min for three exercise intensities in two exercised groups.

As shown in Table 2, the mean of 1MT in the ISOM + EMS group was 18.04 ± 1.02, the means of 60, 70, and 80% of 1MT were estimated as 10.82 ± 0.61, 12.63 ± 0.71, and 14.43 ± 0.82, respectively. When the ISOM + EMS group was given electrical stimulation according to the estimate values and performed isometric exercise, the RPE scores reported by the subjects had a statistically significant difference compared with the estimates (Table 3). That is, the RPE intensity performed by the subjects in the ISOM + EMS group was mostly higher than the estimated value. In addition, as shown in Table 4, the RPE levels in the ISOM group, which only performed isometric exercise, and the ISOM + EMS group, which performed isometric exercise with EMS mixed, showed that the exercise intensities of the ISOM + EMS group were significantly higher. In particular, these results were evident in 70% and 80% of 1MT.

### Demographic and Anthropometric Characteristics

Seventy-five participants (mean age: 61.0 ± 4.11 years) were randomized into three groups: the CON group (*n* = 25), ISOM group (*n* = 25), and ISOM + EMS group (*n* = 25). There were no significant differences between groups for age, height, and weight before the intervention (Table 1).

As shown in Table 1, none of the pre-intervention variables in the three groups were significantly different: body weight (*p* = 0.304), skeletal muscle (*p* = 0.804), fat mass (*p* = 0.748), fat percentage (*p* = 0.405), and basal metabolic rate (BMR, *p* = 0.446). Over time, significant changes were noted regarding body weight, fat mass, fat percentage, and BMR. Specifically, in the ISOM + EMS group, body weight was significantly decreased, and BMR remarkably increased at post-intervention compared to baseline. In terms of fat percentage, there were statistically significant changes over time in all groups, but the pattern of change was different between the groups. The fat percent in the CON and ISOM groups were increased, whereas that of the ISOM + EMS group was decreased. The fat mass of the CON group also significantly increased after 8 weeks compared to the baseline (Table 5).

**TABLE 5:** Differences and Changes in Body composition and Anthropometric variables.

**Variables (unit)**	**Group**	**Time**		**Difference (Post - Pre)**	**Z of Time^**†**^**	**F of Group^**‡**^**
		**Pre (Baseline)**	**Post**			
Body weight (kg)	CON	64.92 ± 9.84	65.91 ± 9.77	0.99 ± 2.71	–1.723	5.060**
	ISOM	64.20 ± 8.47	63.75 ± 8.02	−0.45 ± 2.86	–0.514	
	ISOM + EMS	60.74 ± 8.12	59.52 ± 6.58	−1.22 ± 2.60	−2.043*	
Skeletal muscle mass (kg)	CON	33.81 ± 6.32	32.96 ± 6.20	−0.85 ± 1.89	–1.787	3.035
	ISOM	33.80 ± 6.84	33.12 ± 6.90	−0.68 ± 2.05	–1.453	
	ISOM + EMS	32.44 ± 5.36	33.54 ± 6.08	1.09 ± 3.39	–1.572	
Fat mass (kg)	CON	28.29 ± 5.09	30.21 ± 6.09	1.92 ± 4.04	−2.113*	5.002**
	ISOM	28.62 ± 4.14	28.86 ± 4.72	0.24 ± 3.93	–0.713	
	ISOM + EMS	27.31 ± 5.22	26.35 ± 4.63	−0.96 ± 3.71	–0.511	
Fat percent (%)	CON	30.17 ± 4.90	34.57 ± 7.27	4.40 ± 6.76	−2.719**	12.086***
	ISOM	31.14 ± 6.18	33.89 ± 6.30	2.75 ± 4.60	−2.624**	
	ISOM + EMS	32.17 ± 5.50	29.23 ± 5.65	−2.94 ± 4.81	−2.422*	
Basal metabolic rate (kcal)	CON	1281.45 ± 144.32	1273.99 ± 139.65	−7.46 ± 43.51	–0.686	2.619
	ISOM	1269.60 ± 143.40	1270.49 ± 140.82	0.89 ± 55.53	–0.013	
	ISOM + EMS	1226.54 ± 125.94	1263.15 ± 137.23	36.61 ± 61.64	−2.704**	

All values are expressed as mean ± standard deviation. ^†^ was analyzed using Wilcoxon signed-rank test for comparison of change over time (pre and post). ^‡^ was analyzed using ranked ANCOVA for comparison of variables between groups (CON, ISOM and ISOM + EMS) with the scores of baseline as covariate. *, **, and *** represent *p* < 0.05, *p* < 0.01 and *p* < 0.001, respectively. CON, control group; ISOM, isometric exercise group; ISOM + EMS, isometric exercise + electrostimulation group.

Post-intervention showed significant differences between the groups for body weight, fat mass, and fat percentage. Namely, body weight, fat mass, and fat percentage of the ISOM + EMS group were significantly lower than those of the CON group (F = 9.736, *p* = 0.003; F = 9.594, *p* = 0.003; F = 17.294, *p* < 0.001, respectively). These variables in the ISOM group were less than those of the CON group and more than the ISOM + EMS group, but their differences were not statistically significant.

### Changes and Differences in Inflammatory Cytokines

As shown in Table 6, inflammatory cytokine biomarkers did not differ between the groups at pre-intervention (IL-6 *p* = 0.996, TNF-α *p* = 0.914, CRP *p* = 0.583, RSTN *p* = 0.512), but there were statistically significant changes over time and between the groups. After 8 weeks, the IL-6 and TNF-α levels in the CON group were significantly increased, whereas those in the ISOM + EMS group were significantly decreased. The levels of IL-6 and TNF-α in the ISOM group tended to decrease, but this was not statistically significant. The levels of CRP and RSTN in the ISOM + EMS and ISOM groups significantly reduced over time. On the other hand, their levels in the CON group significantly increased after 8 weeks.

**TABLE 6:** Differences and changes in biomarkers.

**Variables (unit)**	**Group**	**Time**		**Difference (Post – Pre)**	**Z of Time^**†**^**	**F of Group^**‡**^**
		**Pre (Baseline)**	**Post**			
IL-6 (pg/mL)	CON	16.45 ± 6.17	18.12 ± 4.81	1.67 ± 4.61	−2.814**	20.818***
	ISOM	16.56 ± 4.65	15.82 ± 4.13	−0.74 ± 5.55	–1.117	
	ISOM + EMS	16.23 ± 6.51	10.58 ± 4.84	−5.65 ± 4.75	−4.052***	
TNF-α (pg/mL)	CON	28.23 ± 6.98	35.31 ± 10.64	7.08 ± 8.14	−3.756***	19.635***
	ISOM	28.26 ± 8.77	24.00 ± 6.17	−4.26 ± 11.54	–1.844	
	ISOM + EMS	29.09 ± 11.20	20.86 ± 8.81	−8.23 ± 9.99	−2.841**	
CRP (pg/mL)	CON	31.57 ± 13.64	52.38 ± 11.61	20.81 ± 13.67	−4.376***	79.254***
	ISOM	32.08 ± 8.76	27.91 ± 4.90	−4.16 ± 9.48	−1.978*	
	ISOM + EMS	32.71 ± 9.43	23.91 ± 8.77	−8.80 ± 11.04	−2.841**	
RSTN (ng/mL)	CON	5.62 ± 2.15	8.54 ± 3.24	2.92 ± 4.19	−2.921**	33.331***
	ISOM	5.49 ± 1.69	4.02 ± 1.72	−1.47 ± 3.01	−2.571*	
	ISOM + EMS	5.47 ± 2.37	3.44 ± 1.13	−2.02 ± 1.93	−4.269***	

All values are expressed as mean ± standard deviation. ^†^ was analyzed using Wilcoxon signed-rank test for comparison of change over time (pre and post). ^‡^ was analyzed using ranked ANCOVA for comparison of variables between groups (CON, ISOM and ISOM + EMS) with the scores of baseline as covariate. *, **, and *** represent *p* < 0.05, *p* < 0.01 and *p* < 0.001, respectively. CON, control group; ISOM, isometric exercise group; ISOM + EMS, isometric exercise + electrostimulation group; IL-6, interleukin-6; TNF-α, tumor necrosis factor-α; CRP, C-reactive protein; RSTN, resistin.

Significant differences in these biomarkers were noted between the groups in post-intervention. The levels of IL-6, TNF-α, CRP, and RSTN in the ISOM + EMS group were significantly lower than those of the CON group (F = 44.926, *p* < 0.001; F = 49.971, *p* < 0.001; F = 157.711, *p* < 0.001; F = 73.940, *p* < 0.001, respectively), and their levels were lowest among the groups. The levels of these variables in the ISOM group were lower than those in the CON group and more than the ISOM + EMS group. In the ISOM group, the levels of TNF-α, CRP, and RSTN were significantly lower than those in the CON group (F = 23.377, *p* < 0.001; F = 142.541, *p* < 0.001; F = 39.324, *p* < 0.001, respectively), and the IL-6 levels were significantly higher compared to the ISOM + EMS group (F = 21.457, *p* < 0.001).

### Changes and Differences in Isokinetic Torques of the Knee Joint

As shown in Table 7, there were no significant differences in muscle torques at pre-intervention: PT of hamstrings (*p* = 0.889), PTBW of hamstrings (*p* = 0.881), PT of quadriceps (*p* = 0.878), and PTBW of quadriceps (*p* = 0.549). However, there were statistically significant changes over time and between the groups. After 8 weeks, the PT and PTBW of the hamstrings and quadriceps muscle in the CON group were significantly decreased. In the ISOM + EMS group, they showed a tendency to increase, and the enforcement of PT and PTBW in the hamstrings and PTBW of the quadriceps was statistically significant.

**TABLE 7:** Differences and Changes in Isokinetic torques of knee joint at 60 degree/second.

**Variables (unit)**	**Group**	**Time**		**Difference (Post – Pre)**	**Z of Time^**†**^ (*p*)**	**F of Group^**‡**^ (*p*)**
		**Pre (Baseline)**	**Post**			
PT of Flexor (hamstrings) (Nm)	CON	117.64 ± 29.95	101.60 ± 36.64	−16.04 ± 40.43	−1.973*	8.174**
	ISOM	119.44 ± 50.32	120.32 ± 54.55	0.88 ± 36.06	0.000	
	ISOM + EMS	115.48 ± 38.78	138.32 ± 40.90	22.84 ± 31.40	−3.001**	
PTBW of Flexor (hamstrings) (Nm/kg)	CON	1.84 ± 0.50	1.54 ± 0.50	−0.29 ± 0.69	−2.166*	10.909***
	ISOM	1.86 ± 0.71	1.87 ± 0.75	0.01 ± 0.54	–0.040	
	ISOM + EMS	1.91 ± 0.59	2.35 ± 0.70	0.44 ± 0.54	−3.135**	
PT of Extensor (quadriceps) (Nm)	CON	139.64 ± 38.23	120.60 ± 31.61	−19.04 ± 29.87	−2.915*	7.269**
	ISOM	144.28 ± 35.28	140.16 ± 41.71	−4.12 ± 34.86	–1.157	
	ISOM + EMS	138.96 ± 35.57	148.96 ± 29.26	10.00 ± 27.21	–1.829	
PTBW of Extensor (quadriceps) (Nm/kg)	CON	2.13 ± 0.38	1.82 ± 0.37	−0.31 ± 0.46	−3.027**	18.410***
	ISOM	2.25 ± 0.46	2.19 ± 0.55	−0.07 ± 0.50	–1.090	
	ISOM + EMS	2.27 ± 0.40	2.51 ± 0.44	0.24 ± 0.49	−2.327*	

All values are expressed as mean ± standard deviation. ^†^ was analyzed using Wilcoxon signed-rank test for comparison of change over time (pre and post). ^‡^ was analyzed using ranked ANCOVA for comparison of variables between groups (CON, ISOM and ISOM + EMS) with the scores of baseline as covariate. *, **, and *** represent *p* < 0.05, *p* < 0.01 and *p* < 0.001, respectively. CON, control group; ISOM, isometric exercise group; ISOM + EMS, isometric exercise + electrostimulation group; PT, peak torque; PTBW, peak torque per body weight.

At post-intervention, there were strongly significant differences in muscle torques between the groups. The PT and PTBW in the hamstrings (flexor) and quadriceps (extensor) in the ISOM + EMS group were significantly higher than those in the CON group (F = 17.382, *p* < 0.001; F = 22.058, *p* < 0.001; F = 14.582, *p* < 0.001; F = 53.142, *p* < 0.001, respectively), and their levels were highest among the groups. The levels of these variables in the ISOM group were lower than those in the ISOM + EMS group but higher than the CON group. In the ISOM group, PTBW in the hamstrings was significantly lower than that in the ISOM + EMS group (F = 9.027, *p* = 0.004), and PTBW in the quadriceps was significantly higher than that in the CON group (F = 6.381, *p* = 0.015).

### Changes and Differences in KOOS Scores

The baseline KOOS score was statistically insignificant in the three groups: pain (*p* = 0.923), symptoms (*p* = 0.713), activities of daily living (ADL) (*p* = 0.074), sports and recreation (SR) (*p* = 0.645), and knee-related quality of life (KQoL) (*p* = 0.235). However, the ISOM + EMS and ISOM groups showed significant improvement in their KOOS scores pre- and post-intervention. In contrast, the KOOS scores in the CON group showed a tendency to decrease symptoms and SR significantly (Table 8).

**TABLE 8:** Differences and Changes in KOOS scores.

**Variables**	**Group**	**Time**		**Difference (Post – Pre)**	**Z of Time^**†**^ (*p*)**	**F of Group^**‡**^ (*p*)**
		**Pre (Baseline)**	**Post**			
Pain	CON	73.82 ± 7.36	73.19 ± 5.05	−0.63 ± 9.78	–0.444	32.822***
	ISOM	72.40 ± 5.82	75.97 ± 2.68	3.57 ± 7.00	−2.354*	
	ISOM + EMS	72.26 ± 4.19	83.29 ± 4.49	11.03 ± 4.96	−4.345***	
Symptoms	CON	74.24 ± 1.63	72.38 ± 1.20	−1.86 ± 1.56	−4.772***	79.878***
	ISOM	73.91 ± 1.49	81.76 ± 4.59	7.86 ± 4.45	−4.319***	
	ISOM + EMS	74.21 ± 1.58	85.60 ± 4.52	11.39 ± 4.88	−4.346***	
Activities of daily living (ADL)	CON	42.84 ± 4.88	41.31 ± 4.69	−1.52 ± 6.12	–1.118	45.075***
	ISOM	40.26 ± 5.05	44.47 ± 1.43	4.21 ± 5.04	−3.459**	
	ISOM + EMS	39.80 ± 5.87	61.00 ± 10.38	21.20 ± 12.55	−4.292***	
Sports and recreation (SR)	CON	26.00 ± 6.29	23.00 ± 3.23	−3.00 ± 6.77	−2.095*	70.483***
	ISOM	25.00 ± 2.89	48.20 ± 14.71	23.20 ± 14.99	−4.385***	
	ISOM + EMS	27.40 ± 18.32	56.80 ± 13.38	29.40 ± 19.70	−4.205***	
Knee-related quality of life (KQoL)	CON	16.25 ± 4.03	10.85 ± 6.55	−5.40 ± 9.06	–1.738	28.587***
	ISOM	18.50 ± 9.28	40.03 ± 31.52	21.53 ± 30.66	−2.488*	
	ISOM + EMS	15.75 ± 11.28	66.83 ± 4.88	51.08 ± 13.01	−4.376***	

All values are expressed as mean ± standard deviation. ^†^ was analyzed using Wilcoxon signed-rank test for comparison of change over time (pre and post). ^‡^ was analyzed using ranked ANCOVA for comparison of variables between groups (CON, ISOM and ISOM + EMS) with the scores of baseline as covariate. *, **, and *** represent *p* < 0.05, *p* < 0.01 and *p* < 0.001, respectively. CON, control group; ISOM, isometric exercise group; ISOM + EMS, isometric exercise + electrostimulation group.

There were markedly significant differences in the KOOS scores between the groups at post-intervention. The scores for all scales (pain, symptoms, ADL, SR, and KQoL) in the ISOM + EMS group were significantly higher than those of the CON group (F = 59.995, *p* < 0.001; F = 152.625, *p* < 0.001; F = 60.405, *p* < 0.001; F = 103.038, *p* < 0.001; F = 121.297, *p* < 0.001, respectively), and their scores were highest among the groups. The scores in the ISOM group were less than those in the CON and ISOM + EMS groups. In the ISOM group, the scores for pain, symptoms, and ADL were significantly lower in comparison to the ISOM + EMS group (F = 46.015, *p* < 0.001; F = 7.647, *p* = 0.00; F = 57.665, *p* < 0.001, respectively). The scores of symptoms, ADL, SR, and KQoL were significantly higher compared to the CON group (F = 122.380, *p* < 0.001; F = 11.087, *p* = 0.002; F = 176.396, *p* < 0.001; F = 11.248, *p* = 0.002, respectively).

## Discussion

In the present study, we examined whether the eight-week isometric exercise combined with WB-EMS improves serum cytokine levels, muscle strength, and knee function in elderly women with early knee OA. Our study noted that the eight-week isometric exercise program significantly increased the KOOS scores on several subscales (pain, symptoms, ADL, SR, and KQoL) in elderly women with early knee OA. Our results suggest that an eight-week isometric exercise may be beneficial for people with early knee OA by relieving knee pain, alleviating symptoms (such as swelling and restricted range of motion), and improving knee function. Further, our study demonstrated that combined isometric exercise with WB-EMS produced best over-all clinical improvement in OA function as well as decreased levels of inflammatory biomarkers.

Pain and functional impairment in patients with OA reduce their quality of life (Baker and McAlindon, 2000; Abolhasani et al., 2019). It is reported that pain and disability are associated with muscle weakness (van Baar et al., 1998; Villafañe, 2018). Specifically, reduced muscle strength and endurance are found in patients with knee OA (van Baar et al., 1998). These patients show reduced force-generating ability in the quadriceps muscle consequently decreasing strength (peak torque generation) that is important in their physical function (Alnahdi et al., 2012). Such muscular impairments in patients with OA are not limited to the quadriceps but also involve the hamstrings. Knee OA may exhibit muscle weakness without clinical signs of muscle atrophy. Consequently, decreased personal confidence, performance, and independence in daily activities as well as instability are suffered by the patients (Emrani et al., 2006). Reports have shown that strengthening the muscles associated with the knee joint through proper and targeted exercise regimens significantly reduces knee pain and improves knee function (Anwer and Alghadir, 2014). International guidelines, including the American Academy of Orthopedic Surgeons (AAOS), the American College of Rheumatology (ACR), and the Osteoarthritis Research Society International (OARSI) recommend various targeted strengthening, aerobic, stretching, and flexibility exercises for people with knee OA (Kan et al., 2019).

There are three types of basic therapeutic exercises, namely isotonic, isokinetic, and isometric. Among these, isometric exercise is considered to be the most appropriate and easiest for patients to understand. This exercise can be easily and safely performed at home because it requires no or minimal apparatus (Anwer and Alghadir, 2014). However, isometric exercise is reported to be less effective than isokinetic exercise for gaining strength and relieving pain in patients with knee OA (Rosa et al., 2012). Patients who undergo WB-EMS training are believed to have better outcomes, such as the ability to perform maximum isometric knee extension, better functional lower extremity strength, higher fat-free mass, faster gait speed, and reduced risk of falls than those receiving conventional resistance exercises (de Oliveira et al., 2021).

In our study, we also showed that the isometric exercise combined with WB-EMS significantly enhanced the strength of the hamstrings and quadriceps muscles in elderly women with early knee OA compared to controls. We also demonstrated that this combination therapy was superior to isometric exercise alone. The reason is thought to be that, as shown in Table 4 analyzed in this study, exercise intensity is higher when electrical muscle stimulation is combined with isometric exercise than when isometric exercise alone. It was found that the increased amount of exercise through WB-EMS can more effectively improve not only the body composition, but also the muscle function and daily life functions related to the knee joint. Although the PT and PTBW in the muscles of the ISOM group showed a tendency to increase compared to the CON group, it was not statistically significant (Table 7). Along with the enhancement of muscle strength, isometric exercise combined with WB-EMS was more effective than isometric exercise alone in alleviating knee pain and improving knee function as demonstrated by the KOOS questionnaire. Laufer et al. (2014) also reported similar results supporting the addition of neuromuscular electrical stimulation to an exercise program enhanced the positive treatment effects in terms of decreased knee pain immediately after treatment. However, they suggested that the addition of neuromuscular electrical stimulation to an exercise offers only short-term benefits. The effects of neuromuscular electrical stimulation on pain disappeared on the twelfth follow-up week. They attributed this finding to two primary factors: insufficient current intensity or repetitions applied during stimulation and the level of severity of quadriceps muscle insufficiency. Moreover, they used a specially designed chair for electrical stimulation. In their study, participants were seated in a chair and received neuromuscular electrical stimulation (Laufer et al., 2014). Possibly, EMS was insufficiently delivered throughout the entire body.

The mechanism of WB-EMS is same as classical neuromuscular electrical stimulation (NMES). However, unlike NMES, WB-EMS can use several electrodes at the same time and be positioned in different muscle groups to cover more comprehensive area (up to 2,800 cm^2^) (Kemmler and von Stengel, 2013). It is reported that WB-EMS acts directly toward the synthesis of skeletal muscle proteins and is faster than conventional techniques (de Oliveira et al., 2021). NMES is reported to be an effective therapy for quadriceps strengthening in elderly people with knee OA (Bax et al., 2005; de Oliveira Melo et al., 2013). Although the mechanisms of neuromuscular electrical stimulation remain still unclear, it has been reported to increase the electrical activity and muscle mass of the quadriceps (de Oliveira Melo et al., 2016). In the present study, the isometric exercise combined with WB-EMS slightly increased the muscle mass after 8 weeks, but it was not statistically significant. Despite considerable enhancement of PT and PTBW in the quadriceps and hamstrings, isometric exercise combined with WB-EMS did not significantly increase the muscle mass (Tables 5, 7). However, it is also reported that quantitative changes in muscle mass were not sufficient to explain the strength (torque) loss after knee OA (Gür and Cakin, 2003).

Aging is associated with a chronic low-grade inflammatory state characterized by higher circulating levels of inflammatory biomarkers, as seen in OA (Otterness et al., 2000; Franceschi, 2007; Santos et al., 2011). Increased levels of IL-6, TNF-α, and CRP are used to predict chronic low-grade inflammation accompanied by aging (Petersen and Pedersen, 2005; Santos et al., 2011). In OA, cartilage destruction resulting from chondrocyte activation stimulates the release of matrix metalloproteinases (MMPs), disintegrin, and pro-inflammatory cytokines, including IL-6 and TNF-α (Robinson et al., 2016). Thus, people with knee OA have elevated circulating inflammatory mediators such as IL-6, TNF-α, and CRP (Spector et al., 1997; Stannus et al., 2010).

Typically, IL-6, the first cytokine present in the circulation during exercise, increases exponentially in response to exercise, then declines in the post-exercise period. Although exercise remarkably increases the level of IL-6, no studies have reported muscular damage (Petersen and Pedersen, 2005). In general, IL-6 plays an important role in maintaining homeostasis. However, dysregulated, excessive, and persistent levels of IL-6 cause pathological conditions, including acute systemic inflammatory response syndrome and chronic immune-mediated diseases (Tanaka et al., 2018). In patients with OA, higher concentrations of pro-inflammatory cytokines are involved in higher pain levels and worse functional capacity (Penninx et al., 2004). In the present study, the eight-week isometric exercise demonstrated a tendency to decrease the levels of inflammatory cytokines (IL-6, TNF-α, and CRP) and significantly reduced the levels of TNF-α and CRP in elderly women compared to those in the CON group. Furthermore, the isometric exercise combined with WB-EMS was more effective in reducing the levels of these inflammatory cytokines than isometric exercise alone. The ISOM + EMS group had the lowest levels of IL-6, TNF-α, and CRP in our study. In contrast, the participants in the CON group had significantly increased levels of these inflammatory cytokines after 8 weeks (Table 6).

High levels of IL-6 and TNF-α in patients with metabolic syndrome are associated with truncal fat mass (Pedersen et al., 2003). IL-6 and TNF-α are produced in the adipose tissue and inhibit lipoprotein lipase (Coppack, 2001). Adipose tissue and altered lipid metabolism are also involved in increased systemic inflammation, which contributes to knee OA and increased mechanical load (Fain, 2010; Thijssen et al., 2015). In our study, the level of RSTN in the CON group was significantly increased after 8 weeks with an increase in inflammatory cytokine levels including IL-6, TNF-α, and CRP. Alternatively, isometric exercise significantly reduced the RSTN levels compared to the CON group. When combined with WB-EMS, RSTN levels were more effectively reduced (Table 6). RSTN is an adipocyte-specific hormone and has been found to induce the expression of cytokines and chemokines in human articular chondrocytes (Zhang et al., 2010). RSTN upregulates inflammatory chemokines and cytokines in chondrocytes (Jamaluddin et al., 2012). With the reduction of RSTN related to adipose tissue, the combination of isometric exercise and WB-EMS significantly reduced the fat mass and fat percentage. Participants in the CON group exhibited significantly increased fat mass and fat percentage after 8 weeks. Participants in the ISOM + EMS group showed significantly reduced body weight but were slightly increased in the CON group after 8 weeks. Obesity is associated with the incidence and progression of OA. It is also recognized as a risk factor for knee OA (King et al., 2013). A meta-analysis reported that obese people are 2.63 times more likely to develop OA compared to non-obese people (Blagojevic et al., 2010). Thus, weight loss can contribute to significant improvement in pain and delay the progression of structural joint damage (King et al., 2013).

This study was carried out under COVID pandemic. COVID-19 home confinement negatively affected all physical activity including the intensity levels. Moreover, daily sitting time increased, and food consumption and meal patterns including the type of food, snacks between meals and number of main meals were more unhealthy during confinement (Ammar et al., 2020). Reduced physical activity and increased sitting time may increase appetite, overconsumption of food and weight gain through reducing the effectiveness of the “gravitostat” model in which osteocytes may be capable of detecting changes in body mass and appetite in order to maintain a set body weight (Jansson et al., 2018; Ohlsson et al., 2020). In the present study, CON group showed the increased body weight, fat mass and fat percent, and decreased skeletal muscle, although it was not statistically significant. Therefore, special situation called COVID pandemic restricting physical activity may further promote the effect of isometric exercise alone and in combination with WB-EMS in the elderly with early knee OA.

In the present study, the 8-week isometric exercise seems to be an effective non-pharmacological treatment method that significantly alleviated the pain and symptoms and improved knee function in elderly women with early knee OA. This contributed to a significant improvement in their daily life. However, exercise alone did not significantly enhance muscle strength and/or reduce the levels of inflammatory cytokines. Interestingly, when combined with WB-EMS, the beneficial effects of isometric exercise on early knee OA notably increased. Isometric exercise combined with WB-EMS significantly enhanced the strength of the quadriceps and hamstring muscles and reduced the levels of various inflammatory cytokines including IL-6, TNF-α, CRP, and RSTN. Furthermore, this combination effectively alleviated knee pain and symptoms, including swelling and restricted range of motion. The subsequent amelioration of knee function resulted in improvement in the quality of life of elderly women with early knee OA. It was beneficial for weight loss by reducing fat mass and fat percentage. These results highlight the multiple benefits of combined isometric exercise in elderly patients with early knee OA in terms of pain relief, functional improvement, and stronger muscles.

The primary goal of knee OA intervention is to reduce knee pain and physical disability and to improve functional capacity. Therefore, our findings recommend effective interventions for knee OA patients given the limited available literature on the combination of isometric exercise and WB-EMS. These non-pharmacologic, non-invasive interventions should be considered by healthcare specialists for their elderly patients with early knee OA. However, there are some limitations to our study. First, this is a pilot study so our results should be judiciously interpreted and considered. The small sample size used in the present study might decrease the interpretability and generalizability of our results. The longer intervention period, the better the intervention effect may be. Second, we also did not investigate the effect of EMS alone on knee OA. Third, there was no control for medications administered to all participants, such as anti-inflammatory drugs. Finally, other biomarkers involved in knee OA should be examined and identified to evaluate the effect of isometric exercise and WB-EMS. For example, leptin and adiponectin are reported to affect OA through direct joint degradation or local inflammatory control (Sowers and Karvonen-Gutierrez, 2010). Future studies should increase the sample size, control medication usage of participants, and consider the appropriate intervention time. It is also necessary to examine and measure various biomarkers associated with knee OA.

## Data Availability Statement

The raw data supporting the conclusions of this article will be made available by the authors, without undue reservation.

## Ethics Statement

The studies involving human participants were reviewed and approved by 2-1040781-A-N-012021024HR. The patients/participants provided their written informed consent to participate in this study.

## Author Contributions

S-HP and Y-SJ conceived the idea and verified the methods section. SM and JY developed the background and performed the calibration of different devices used in the tests. S-HP performed the tests. Y-SJ wrote the manuscript with support from S-HP. SM and S-HP contributed to the interpretation of the results and data analysis, drafted the manuscript, and designed the figures and tables. All authors provided critical feedback, helped shape the research, analysis, and manuscript, discussed the results, and contributed to the final version of the manuscript.

## Conflict of Interest

The authors declare that the research was conducted in the absence of any commercial or financial relationships that could be construed as a potential conflict of interest.
